# Public Health and International Partnerships in the Democratic People’s Republic of Korea

**DOI:** 10.1371/journal.pmed.1001929

**Published:** 2015-12-29

**Authors:** John Grundy, Beverley-Ann Biggs, David B. Hipgrave

**Affiliations:** 1 School of Health and Social Development, Deakin University, Burwood, Victoria, Australia; 2 The University of Melbourne, Department of Medicine (Royal Melbourne Hospital) at the Doherty Institute, Parkville, Victoria, Australia; 3 The Victorian Infectious Diseases Service, Royal Melbourne Hospital, Parkville, Victoria, Australia; 4 Nossal Institute for Global Health, Melbourne School of Population and Global Health, The University of Melbourne, Parkville, Australia

## Abstract

David Hipgrave and colleagues argue that sustained collaboration is required to improve population health and health services in North Korea.

**Summary Points**

- The health system in the Democratic People’s Republic of Korea (DPRK) is suitable for high public health program coverage, with a wide facility network and high staff-population ratios.
- Economic difficulties, natural disasters, and poor resourcing of the health sector in the 1990s had catastrophic impacts on public health and system functioning, leading to sharp declines in vaccination coverage.
- With considerable international support, diphtheria, tetanus, and pertussis (DTP) vaccine coverage has risen from 37% in 1997 to 96% in 2013. Major challenges related to immunization services and the health sector more generally have been reduced.
- This recovery demonstrates the potential for international partners to support DPRK’s national health agencies and improve public health programming, notwithstanding tensions in international relations and challenging domestic conditions.
- Sustained collaboration is required to improve population health and health services in DPRK. This has regional and global public health implications and may influence ongoing political tensions.

## An Overview of the Public Health Situation in DPRK

The Democratic People’s Republic of Korea (DPRK, or North Korea) has faced formidable public health challenges in recent decades. In the mid-1990s, a crisis developed in the context of complex national and international circumstances, with deteriorating health care and public health program coverage, food insecurity, and widespread malnutrition despite large-scale international food aid. Life expectancy fell from 71 to 69 years from 1990 to 2010. The under-five mortality rate (U5MR) rose from 45 per 1,000 live births in 1990 to 58 in 2000 [1]. Maternal mortality is estimated to have risen over 40% in the same decade [2]. Outbreaks of vector-borne and other communicable diseases continued into the early 2000s, including around 140,000 cases of vivax malaria in 2001 [3]. The number of tuberculosis cases was high, but reporting was evidently poor in the 1990s [4]. External agency estimates of diphtheria, tetanus, and pertussis (DTP) vaccine coverage fell below 40% during this period [5].

Grave concerns continue to be expressed in regards to DPRK’s health system [6,7]. Although notification rates are improving, tuberculosis incidence in 2012 (409 per 100,000 people) and prevalence (511 per 100,000) remained very high compared to regional averages of 187 and 264, respectively [4]. Multidrug resistance is problematic [8,9]. Child undernutrition also remains a major public health concern; a 2012 population-based survey of 8,040 children under the age of five years in all ten provinces identified stunting among 27.9% of those aged 12–23 months, as well as stunting of 36.8% and underweight of 15.2% among those aged 24–35 months. Among children aged 6–59 months, 28.7% were anaemic, implying widespread iron deficiency, with serious implications for child development [10]. The United Nations Children's Fund (UNICEF) figures on child undernutrition in DPRK are marginally worse than this survey data, with rates of stunting (32%) and underweight (19%) far higher than those in DPRK’s East Asian and Pacific neighbours [11]. Moreover, there is a high degree of vulnerability to food insecurity due to environmental shocks, and dependence on external food aid [12]. A high prevalence of iodine deficiency was also detected among 1,200 children assessed across DPRK in 2010 [13]. Notwithstanding these problems, the reported quality of these and other surveys demonstrated encouraging openness to stratified and internationally verified assessments.

With the exception of food aid, DPRK has been challenged by very low volumes of international and domestic support for the national health system. Development assistance for DPRK in 2013 was much lower than it was for many nearby and regional developing nations (Table 1), and limited support has been provided by nongovernmental organizations (NGOs) and UN agencies since the mid-1990s [14,15]. Although the GAVI Alliance, Global Fund, and international NGOs have recently funded immunization services and malaria, tuberculosis, and maternal and child health programs, <45% of the critical UN agency humanitarian programs in DPRK were actually funded in both 2013 and 2014, with severe cuts to food aid as a result of insufficient funds for the World Food Program [16].

**Table 1:** Net receipt of overseas development assistance (ODA) per capita in 2013 in selected nations.

	Net receipt of ODA in US$ millions 2013	Population (millions) 2013	ODA per capita 2013
DPRK	30	24.9	1.21
Vietnam	2,381	91.7	25.97
Cambodia	535	15.1	35.47
Laos	268	6.8	39.43
Myanmar	3,603	53.3	67.60
Timor Leste	200	1.1	182.33

Sources: https://stats.oecd.org/qwids/ and UN Population Fund (UNFPA) State of the World’s Population Report 2013, available at www.unfpa.org

Published reports on public health in DPRK are uncommon, but recent planning and financial sustainability exercises, population-based surveys, and other reports, all available online, indicate recovery of its vaccination program [5,17] and high coverage and good treatment outcomes of its activities in tuberculosis control [9,18]. They suggest that despite major challenges, international collaborations have contributed significantly to this progress and indicate the importance of such partnerships for equitable improvements in public health and reducing global health risks regardless of other circumstances. This is particularly evident for child vaccination services, which are a focus of this report.

## Sources of Information on Health and Public Health Programs in DPRK

Multilateral organisations, international NGOs, and academic groups participate in planning and financial analysis exercises, population-based surveys, vaccination campaigns, external funding applications, and interaction with development organizations in DPRK. Reports and related documentation in the public domain include global databases such as those published by UNICEF, the World Bank, WHO, and the United States government and national surveys conducted by the government with external support [10,17,19,20], successive immunization plans covering 2007–2015 [5,21], and various project evaluations, assessments [9,22,23], and proposals [20,24–26]. For this analysis, we used these and similar documents providing publicly available information on maternal and child health and nutrition in DPRK. In addition, we drew on the limited academic literature on health in DPRK. A search in November 2015 using the PubMed-National Center for Biotechnology Information database (http://www.ncbi.nlm.nih.gov/pubmed) and title/abstract search terms “Democratic People’s Republic of Korea” OR “DPRK” OR “North Korea” with no date limit identified 338 citations (up from 221 in early 2013), but very few of these reported population-based data.

## Health Services in DPRK

The DPRK health system serves a population of ~24.5 million, with an estimated annual birth cohort of 350,568 [20,27]. It was established in the early 1960s and designed for high population coverage. A network of facilities includes 133 hospitals and 6,233 primary care “*Ri*” clinics, with 215,727 health staff [17,28]. The ratio of health human resources to population (7.6 per 100,000) is among the region’s highest [5], although the quality of personnel has not been independently assessed. Health workers receive basic training that emphasises textbook learning rather than acquisition of competency; there is limited supervision or access to updated knowledge, technologies, and practices [29].

“Section” or “household” doctors at the *Ri* clinics are each assigned to ~130 dwellings and are responsible for clinical and public health services, ensuring a high degree of surveillance and directly observed treatment capacity for diseases such as tuberculosis. *Ri* clinics link local communities to the national health system and are managed by county hospitals in DPRK’s 208 counties, which are themselves overseen by province-level referral facilities.

Despite high coverage, DPRK’s health system has many shortcomings, including aging infrastructure, lack of transport, irregular electricity, heating, and water supply, lack of quality medicines and other equipment, and limited operational budget [19,30]. Although institutional delivery approached 95%, maternal mortality was estimated at 87 per 100,000 live births in 2013 [2]. Among 267 maternal deaths reported in 2009, more than a third occurred in health facilities, suggesting problems with quality of care [20].

Inequity prevails in health service delivery and access and in health outcomes. In 2010, the government reported that only 30% of counties had adequate transport and communications equipment to operate a referral system [5]. The 2012 National Nutrition Survey demonstrated modest reductions in stunting since 2009 but wide geographic variation; severe stunting ranged from 4% in the capital Pyongyang to 12% in north-eastern Ryanggang province [10,17,31]. Wide disparities in anaemia rates, iodine deficiency disease risk, and vaccination coverage also prevail [10,13,19]. Other analyses highlight widespread shortages in the availability of essential medicines [29]. A recent planning exercise for DPRK’s health sector indicated a 67% funding deficit for implementation of priority basic services [5]; the government funded only 21% of routine vaccines in 2010 [32].

## The Immunization System in DPRK

DPRK’s expanded program on immunization (EPI) was established in the early 1980s. However, domestic vaccine production fell sharply, and UNICEF has supplied all vaccines for DPRK’s EPI since 1995. A network of hygiene and anti-epidemic institutes oversees vaccination, vaccine-preventable disease surveillance, and outbreak response from the central to the county level [21]. A hierarchical logistics and warehousing system is maintained [5,22], and the EPI is implemented by *Ri* clinic doctors according to a WHO-recommended schedule [5].

### Vaccination Coverage and Vaccine-Preventable Disease Control: 1980–2011

**Fig 1:**
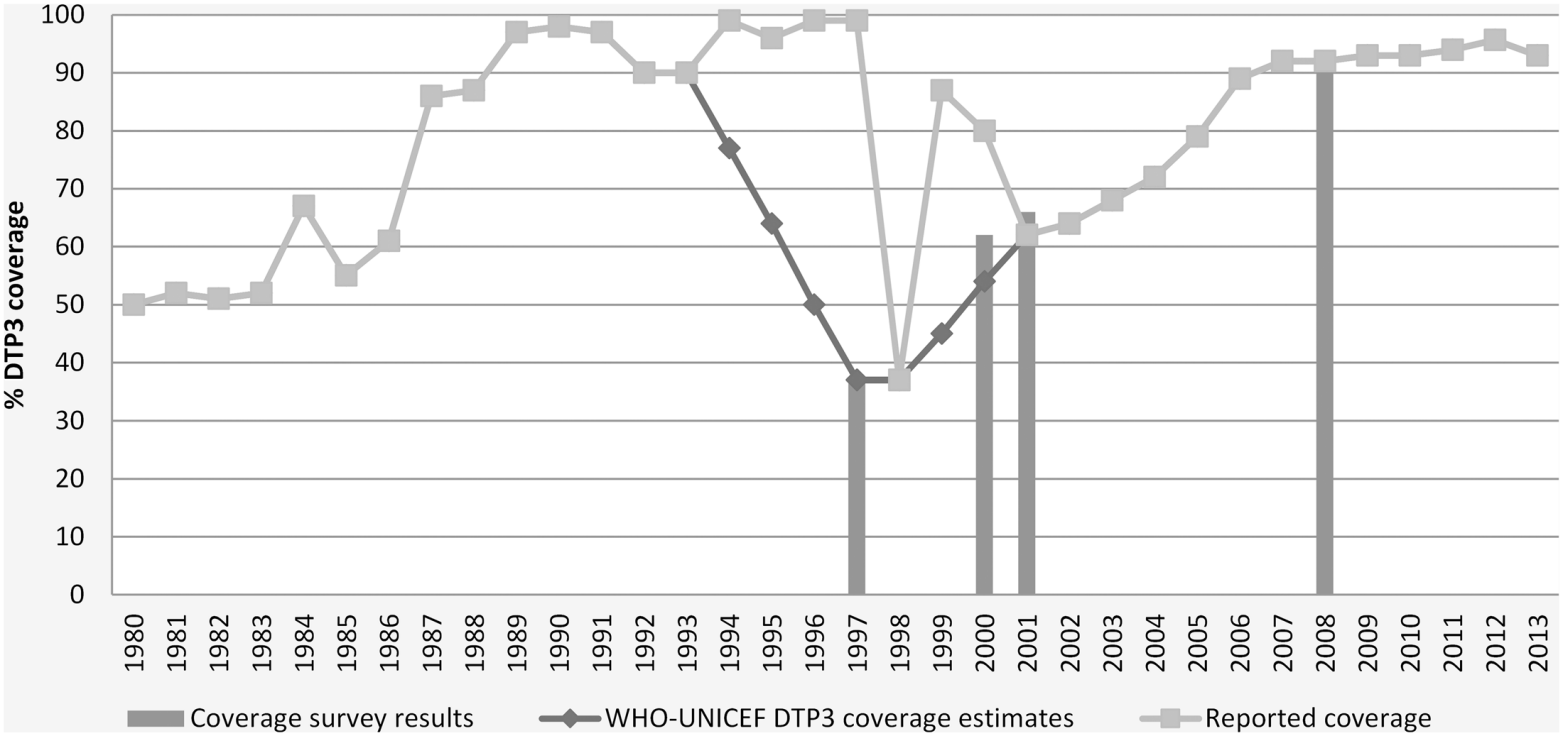
WHO/UNICEF estimates of DTP3 coverage in DPRK, 1980–2013 [34].

Following a gradual increase in the 1980s and a steep decrease in the 1990s, substantial improvements to vaccination coverage occurred in the last decade [5,33]. Coverage with the third dose of diphtheria-tetanus-pertussis vaccine (DTP3) increased from 37% in 1997 to 96% in 2013. Wide differences between government and UN estimates of DTP3 coverage between 1993 and 2000 exemplified the unreliability of DPRK’s health statistics during the 1990s (also observed for tuberculosis [18]), but coverage estimates have merged since 2001 (Fig 1), when WHO established its office in Pyongyang. During the 2008 national EPI survey, UNICEF and WHO followed the standard protocol and used standardised data collection tools, and the survey team had unrestricted access [19].

In 2010, all 208 counties achieved coverage of >80% for DTP3 [34]. Improvements were also noted for other antigens, including measles and polio vaccines, and an estimated 91% of newborns were protected at birth from tetanus in 2010 (compared to 60% in 1997) [34]. No cases of neonatal tetanus were reported in the decade to 2009 [17,20], and DPRK has been polio free since 1996 [5]. However, a measles outbreak in 30 counties in 2007 provoked a nationwide campaign supported by WHO, UNICEF, the Red Cross, and the government and the addition of a measles booster at age 15 months to the EPI in 2009 [5]. In addition to the hepatitis B vaccine in 2003, DPRK introduced the pentavalent vaccine in mid-2012, supported by UNICEF, WHO, and the GAVI Alliance. Internationally supported and observed campaigns saw approximately 4.5 million children vaccinated with Japanese encephalitis vaccine between 2009 and 2014 [35,36].

### Challenges to Vaccination Services in DPRK

In more difficult aspects, DPRK’s EPI remains weak. While it has achieved reporting standards for polio and aspects of measles surveillance, low reporting of adverse events and inadequate subnational disaggregation of vaccination coverage reporting and surveillance suggest that the surveillance network only operates at a basic level.

Difficult roads and terrain, unreliable energy supplies, and severe weather are also important challenges. A nationwide cold chain assessment in 2008 identified a system in disrepair, with 40% of vehicles out of order and <14% of refrigerators having thermometers [22]. The cold chain was subsequently completely rehabilitated with external funding and technical support. A follow-up assessment in 2011 [37] reported significant improvement, but persisting equipment shortages and transport constraints. GAVI and UNICEF recently provided vehicles for all counties.

### Financial Sustainability and the Importance of International Support

**Fig 2:**
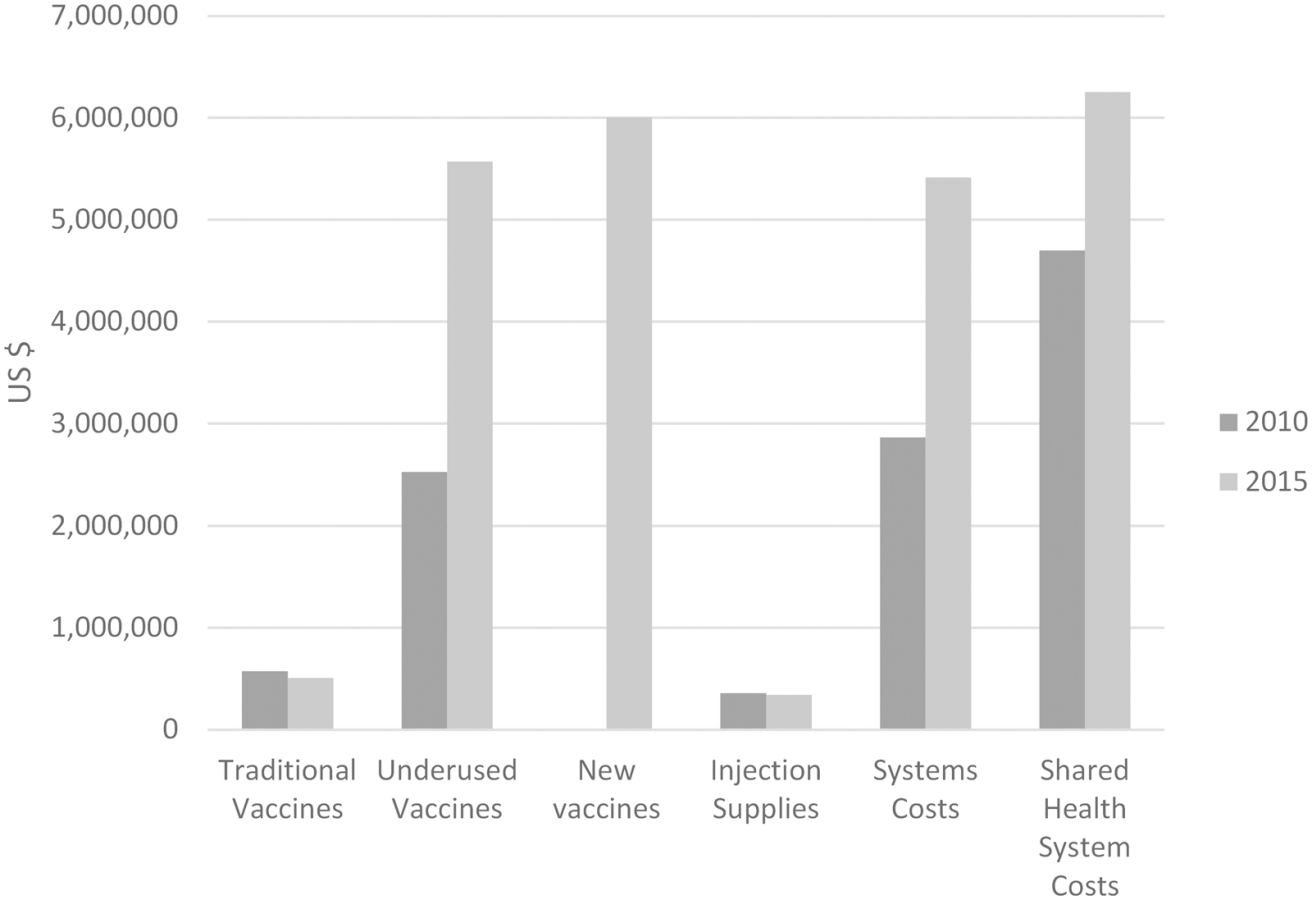
DPRK baseline immunization budget in 2010 and forecast expenditures in 2015 [21].

The campaigns and improvements to DPRK’s EPI since 1996 have been mostly supported by international partnerships (Table 2). The pentavalent vaccine and planned introduction of measles-mumps-rubella and rotavirus vaccines will further increase the national EPI budget (Fig 2), increasing DPRK’s reliance on external support [38]. Despite increased government inputs, the percentage of the program financed domestically will decrease from 28% in 2010 to an estimated average of 12% during the period of 2011–2015 [5]. Clearly, the improvements to DPRK’s EPI will require ongoing international and national partnership.

**Table 2:** Timeline of international support for and achievements in immunization in DPRK, 1996–2012.

Year	Achievement
**1996**	Commencement of UNICEF and WHO support for vaccines, cold chain equipment, transport, technical assistance, and human resource capacity building. NGO assistance for health commenced in the mid-1990s.
**2001**	Commencement of partnership with GAVI
**2003**	Development of Financial Sustainability Plan for immunization
**2003**	Introduction of hepatitis B (HepB) vaccine
**2006**	Multiyear plan for immunization in 2006
**2007**	Achievement of polio eradication certification
**2007**	Successful nationwide measles campaign following an outbreak
**2008**	Introduction of measles vaccine second dose in 2008 through GAVI support
**2008**	National surveys for cold chain and EPI coverage conducted with support from UNICEF
**2009**	Revised cold chain policy and national immunization schedule
**2009**	Commenced government cofinancing for the tetravalent vaccine (DTP-HepB) from GAVI
**2010**	Extension of cold chain system to county level through UNICEF and GAVI support
**2009–2010; 2013–2014**	Implementation of nationwide Japanese encephalitis campaigns covering 4.5 million eligible children
**2011**	Multiyear plan for immunization (2011–2016) with technical support from WHO and UNICEF
**2011**	Zero measles cases reported (2008–2011)
**2011**	Increase in immunization coverage (DTP3) from 37% in 1997 to 94% in 2011
**2012**	Introduction of *Haemophilus influenza* type b vaccine (GAVI and government cofinancing)

Adapted from information in GAVI and the Ministry of Public Health in the Democratic People’s Republic of Korea (2011) Comprehensive Multi Year Plan for Immunization (2011–2015) [5].

Nonetheless, such support seems imperative. Vaccination may have contributed to the decline in DPRK’s U5MR from 58 in 2000 to 33 in 2011 [1]; measles and tetanus no longer contribute significantly to child mortality. Among the estimated 11,735 under-five deaths in 2010, 12.7% were attributable to pneumonia and 5.3% to diarrhoea, suggesting that new vaccines recently introduced and in planning will contribute to further declines in child mortality [39].

## The Benefits and Risks of International Health Partnerships with DPRK

The available evidence suggests that international support has substantively improved public health in DPRK, openness to international oversight, and application of global public health standards.

Since the 1990s, vaccine coverage increased dramatically, and sustained declines of vaccine-preventable disease have been achieved. While resource constraints continue to affect the routine EPI, the health facility network has effectively implemented internationally supported vaccination campaigns as recently as 2014 [36]. The merging of coverage estimates in the last decade and cooperation with standard survey procedures indicate acceptance of global standards for measuring performance. The extensively revitalized cold chain system increases the likelihood that effective vaccines are reaching children. The recently introduced and planned new vaccines [5] will protect children against additional causes of under-five mortality and disease. Health system strengthening initiatives are also supporting decentralized EPI planning.

These improvements in vaccination services have occurred in conjunction with other public health improvements in DPRK, again assisted by international partnerships. International support has yielded a sharp reduction in malaria, to around 16,000 cases in 2012 [3]; DPRK is now targeting pre-elimination of the disease [40]. It has also yielded higher tuberculosis detection and treatment completion rates and reduced tuberculosis mortality rates [4,9]. The observed 10% decline in child stunting between 2009 and 2012 [10,17,31] was possibly due not only to improved harvests but also to agricultural inputs, distribution of fortified food in nurseries and to pregnant and lactating women, micronutrient supplementation for women and children, improved access to health services, and food aid—again all undertaken with international support [10,23].

However, these improvements must be balanced against persisting constraints and resulting risks. Notwithstanding the social and public health gains attributable to declines in communicable diseases, improved public health in DPRK is tenuous [41]. Ongoing international tensions, comprehensive sanctions, adverse domestic and international economic conditions, and constraints to modernization of the agricultural and manufacturing sectors limit the government’s income stream and capacity to cofinance program costs. As GAVI Alliance and Global Fund support declines, ongoing support from other development partners and increased government cofinancing will be essential for sustained population access to vaccination services and other public health interventions. UN programs also require major new funding, with a shortfall of US$93 million in 2015 [15].

This perspective makes assumptions because of the limited amount of independent verification; weak surveillance and the lack of administrative and other data from subnational levels remain significant challenges to the nation’s health system as a whole. Nevertheless, the publication of census data over the last 10–15 years [20] and the execution of population-based assessments such as UNICEF’s multi-indicator cluster and the other surveys described [10,13,17,19], all overseen jointly by government and international agencies, build confidence in understanding the health needs of DPRK’s population and the likely impact of internationally supported public health interventions. While concerns may be raised about the sampling, representativeness, and independence of UN-agency-sponsored surveys, nothing in those published indicates that they are substantively less reliable than similar work in many other developing countries. On this basis, cautious conclusions, along with inferences for regional public health, can be drawn.

DPRK’s circumstances are similar to those observed previously in countries such as Myanmar [42], Cambodia [43], and much earlier in China [44], where long-term investment was required to build or rebuild health systems as well as trust and confidence in international support. Work in Nepal [45] and a review of aid effectiveness in other fragile states have emphasized the value of partnerships over the long term [46]. Moreover, notwithstanding the constraints placed on the activities of external organisations in DPRK (almost certainly contributing to low development assistance funding), the evident increasing openness for external involvement in program oversight and disaggregated surveys suggest untapped potential for additional collaboration between DPRK’s national health agencies and international partners. Public health in DPRK, and globally, will benefit from such engagement and should not be held hostage by political considerations and security concerns; both international and domestic agencies should consider the health and humanitarian benefits of increased partnerships in DPRK [47]. The evidence presented suggests that international partnerships can positively influence the DPRK government in ways that isolation and political brinksmanship may not. All will benefit from a more collaborative environment.

Moreover, careful consideration of the demonstrated benefit of international support for public health may broaden the international discourse on DPRK beyond its almost exclusive focus on state security, towards the social security question of local and regional public health. Heightened attention to issues of equity and public health in DPRK may serve to not only enhance partnerships for health but also to support longer term peace and development.

## Abbreviations

DPRK: Democratic People’s Republic of Korea
DTP: diphtheria, tetanus, and pertussis
DTP3: third dose of diphtheria-tetanus-pertussis vaccine
EPI: expanded program on immunization
HepB: hepatitis B
NGO: nongovernmental organization
ODA: overseas development assistance
U5MR: under-five mortality rate
UN: United Nations
UNFPA: United Nations Population Fund
UNICEF: United Nations Children's Fund
